# Task sharing for point-of-care testing: Review of national health policies and implementation landscape in 19 African countries

**DOI:** 10.1371/journal.pgph.0005485

**Published:** 2025-12-29

**Authors:** Zibusiso Ndlovu, Mariamo Ibraimo Assane, Francis Ocen, Ki-zerbo Charles Lamou, Musonda Mandona, Fingani Mphande-Nyasulu, Emmanuel Kabwe, Noah Takah Fongwen, Halifa Mbae Said, Omolara Emmanuel, Emeka Elom, Sandra Chipuka, Susan Nabadda, Joseph Bitilinyu-Bangoh, Grace Lesuk, Nancy Bowen, Diomandé Adama, Koleka Mlisana, Jaya A. George, Daniel Melese, Gonfa Ayana, Rollin Ndombe, Cheryl Case Johnson, Anne Bekelynck, Pascale Ondoa, Marguerite Massinga Loembé, Tom Ellman, Nqobile Ndlovu, Geoffrey Fatti, Collins Otieno Odhiambo

**Affiliations:** 1 Southern African Medical Unit (SAMU), Medecins Sans Frontières, Cape Town, South Africa; 2 Division of Epidemiology and Biostatistics, Department of Global Health, Faculty of Medicine and Health Sciences, Stellenbosch University, Cape Town, South Africa; 3 African Society for Laboratory Medicine (ASLM), Addis Ababa, Ethiopia; 4 Ministry of Health, Lusaka, Zambia; 5 Faculty of Medicine, King Mongkut’s Institute of Technology Ladkrabang, Bangkok, Thailand; 6 Kazan (Volga region) Federal University, Kazan, Russia; 7 Centre for Laboratory Diagnostics and Systems, Africa Centres for Disease Control and Prevention (CDC), Addis Ababa, Ethiopia; 8 National AIDS, Viral Hepatitis and STIs Control Program, Department of Public Health, Federal Ministry of Health and Social Welfare, Abuja, Nigeria; 9 Medical Laboratory Services Division at Nigeria’s Federal Ministry of Health and Social Welfare, Abuja, Nigeria; 10 Ministry of Health and Child-Care, Harare, Zimbabwe; 11 National Health Laboratory and Diagnostic Services, Ministry of Health, Kampala, Uganda; 12 National Public Health Laboratory Services, Ministry of Health and Sanitation, Lilongwe, Malawi; 13 National Health Laboratory Services, Ministry of Health, Juba, South Sudan; 14 Division of National Laboratory Services, Ministry of Health, Nairobi, Kenya; 15 Ministry of Health, Yamoussoukro, Côte d’Ivoire; 16 National Health Laboratory Services, Johannesburg, South Africa; 17 Department of Chemical Pathology, National Health Laboratory Services and University of the Witwatersrand, Wits Diagnostic and Innovation Hub, Johannesburg, South Africa; 18 Ethiopian Public Health Institute (EPHI), Addis Ababa, Ethiopia; 19 Médecins Sans Frontières, Kinshasa, Democratic Republic of Congo; 20 World Health Organization, Geneva, Switzerland; 21 The Global Fund to fight AIDS, Tuberculosis and Malaria, Geneva, Switzerland; 22 Amsterdam Institute for Global Health and Development, University of Amsterdam, Department of Global Health, Amsterdam, The Netherlands; 23 Consultant, Health Systems Strengthening and Diagnostics, Libreville, Gabon; Menzies School of Health Research, AUSTRALIA

## Abstract

World Health Organization recommends task sharing (TS) for point-of-care testing (POCT) with lay health workers (LHW) to improve access when professional capacity is limited. Despite many benefits of POCT, TS remains underutilized. This study examined uptake of TS for POCT in national policies and the implementation landscape in 19 African countries from November 2024 to March 2025. A mixed-methods approach included an online cross-sectional survey with stakeholders (national ministries of health, medical associations, private laboratories, implementation supporting partners, LHWs); review of national health strategic and policy documents and key informant interviews (KII) with national laboratory directors. Standardized emails recruited participants, whilst documents came from requests and online searches. Quantitative data were analysed descriptively, and KII data with framework analysis. Of 217 policy documents collected, 197 (91%) were relevant. Over half of national health strategic plans (10/19; 53%) recognize LHWs as vital for expansion of primary healthcare services, but fewer (7/19; 37%) mention TS. While 58% (11/19) of national laboratory strategic plans aimed to expand POCT access and quality, 84% did not mention LHW to support TS. Among national HIV/AIDS strategic plans, 53% (9/17) referenced TS for POCT, mainly for HIV diagnosis; with only one addressing POCT for advanced HIV disease. Outside HIV and malaria, LHW POCT was rarely emphasised in disease-specific strategic plans. Seventy-five stakeholders (67% male) completed the online survey, and six KII were conducted. All reported that LHW conduct POCT, mainly with donor-support. HIV rapid testing was cited as having the most structured training program. National laboratory leaders acknowledged implementation challenges but saw opportunities to expand LHW-led POCT. Shifting from fragmented, disease-specific approaches to multi-disease TS model is crucial for sustainable POCT. Coherent policy and implementation reforms are needed to institutionalize TS amid declining resources. National laboratory leadership should drive the adoption of training and quality assurance for TS for multi-disease POCT.

## Introduction

Point-of-care (POC) testing for the screening, diagnosis and monitoring of diseases, as well as other conditions, is one of the most practical and scalable approaches for public health strategies, as it can overcome key barriers that limit equitable, timely and effective testing [1,2]. POCT encompasses various modalities which include lateral flow assays, handheld devices, near-POC benchtop analysers as well as emerging lab-on-a-chip technologies [3,4]. POCT can facilitate prompt linkage to care, access to therapeutic interventions, enable prompt disease outbreak response, support antimicrobial stewardship and improve overall healthcare efficiency [2,5,6] among other benefits. Despite significant progress in expanding access to simplified diagnostics, studies show that only 19% of the population in low- and middle-income countries (LMIC) have access to the simplest POC tests for healthy pregnancy and non-communicable diseases (NCDs), excluding HIV and malaria tests [7]. The lack of adequate attention given to POC diagnostics by policymakers and funders, combined with shortages of trained personnel for POCT as well-as inconsistent availability of testing supplies, have significantly contributed to this diagnostic gap [8,9]. Modelling studies have shown that reducing the diagnostic gap for six tracer conditions (HIV, hypertension, diabetes mellitus, syphilis, tuberculosis (TB), congenital hepatitis B) could reduce the annual number of premature deaths in LMICs by 1,1 million [7].

Primary health care (PHC) facilities are the first point for seeking healthcare among patients; however, these facilities often lack clinical laboratory services. Laboratory-trained technicians are usually scarce and often not present in these facilities to conduct multi-disease POCT, which is recommended by WHO [10] and included in many national essential in-vitro diagnostic lists (NEDL). In these settings, POC tests - which can aid in the identification of communicable (HIV, tuberculosis, cryptococcal meningitis, malaria, syphilis, hepatitis, cholera, Covid19) as well as NCDs (diabetes), among other conditions- are usually administered by nurses and clinical staff members. These cadre already have patient care responsibilities and may have limited capacity to effectively use POC tests [11]. Assigning POCT responsibilities to these cadre can increase service delivery costs and potentially diminish the overall impact of health services by diverting valuable health worker time away from other critical patient care activities. The use of the repertoire of simplified POC tests, especially in health facilities without on-site laboratories, has not been accompanied by corresponding adjustments in human resources to maximise the impact of POCT.

WHO strongly recommends task sharing (TS) of POCT with non-laboratory personnel [12,13] as a pragmatic response to health care worker (HCW) shortages. TS is the rational collaborative re-allocation of tasks from professional HCWs to trained lay health workers (LHWs) [13,14]. LHW are personnel who perform functions related to healthcare delivery but do not possess formal professional or paraprofessional education. They typically receive training tailored to their specific role determined by the ministries of health; however, they can be a versatile cadre even capable of supporting therapeutic and mental health interventions [15–17], among other roles. Strategies like self-testing have also been adopted in some settings to enable decentralization of testing services to individuals and communities.

Studies have shown that LHWs, with adequate training, can reliably perform POCT services for rapid HIV, malaria, hepatitis, syphilis, urinary *Mycobacterium tuberculosis* lipoarabinomannan (urine TB LAM), cryptococcal antigen, haemoglobin, CD4 cell count and near POC molecular instruments [1,2,18–24]. LHW in the studies included counsellors, health diagnostic assistants, phlebotomists, microscopists, community healthcare workers, and health surveillance assistants; and reasons for TS ranged from HCW shortages to decentralizing access to health services. However, some of these studies also highlight that decentralized task shared POCT requires support from national laboratories and programmes to ensure adequate training, mentorship and quality assurance [9,19]. Other studies have also shown that the implementation of TS for POCT has proven difficult in practice due to lack of supportive national policy and fiscal plans, complex governance for POCT conducted outside laboratories together with strict legal structures which enforce professional boundaries on POCT [9,25]. A national policy review study for HIV testing services across 50 countries revealed that only 42% permitted LHWs to conduct POCT [26].

By 2050, Africa’s population is expected to double to two billion, and this growth, coupled with climate change, urbanization and ecological shifts, is likely to lead to more frequent and severe disease outbreaks and other emerging infections. The rising surge of largely undiagnosed NCDs, now causing 37% of deaths in sub-Saharan Africa [27,28], adds to this burden. This trend underscores the growing need to scale up TS for integrated multi-disease POCT with LHW to expand equitable access to testing, improve integration of disease programs and strengthen public health responses. Scaling TS is also critical to advance global health priorities like universal health coverage and health security, as emphasized by the 2023 World Health Assembly resolution on strengthening access to quality diagnostics [29].

Reimagining TS for POC tests is an important discourse for health programs as TS is one of the potentially sustainable ways to overcoming testing gaps especially in PHC, particularly as national programs are experiencing unprecedented funding challenges [30], yet with high testing demands. This study aimed to review the uptake of TS for POCT with LHW in national health policies and strategic documents as well as its implementation in 19 African countries; to guide future policy and scale-up efforts.

## Methods

### Ethics statement

The study was approved by Stellenbosch University Human Ethics Research Committee (S23/11/298). Potential informants received a standardized email with a link to the online survey tool (APoCTe), explaining the study purpose, risks, and confidentiality. Respondents consented by selecting an online verification button before completing the survey.

This descriptive mixed-methods study used an online cross-sectional survey, virtual key informant interviews (KII) with selected national laboratory leaders, and a review of national strategic and policy documents.

The study was conducted in 19 countries participating in the laboratory systems strengthening community of practice (LabCoP) convened by the African Society for Laboratory Medicine (ASLM): Botswana, Burundi, Burkina Faso, Côte d’Ivoire, Democratic Republic of Congo (DRC), Eswatini, Ethiopia, Gabon, Kenya, Malawi, Mozambique, Nigeria, South Africa, South Sudan, Sierra Leone, Tanzania, Uganda, Zambia, and Zimbabwe.

Study participants were key decision-makers knowledgeable about POCT and or TS with LHWs. Key stakeholder mapping was conducted in October 2024 to guide the identification of potential informants, based on ASLM, Africa CDC and Medecins Sans Frontieres (MSF) contacts in the continent. Purposive sampling was used to identify individuals who occupy key positions within the national health program policy and implementation, vertical disease programs (HIV/AIDS, TB, malaria, NCDs), implementation partners supporting testing services, laboratory and medical councils, public and private laboratory management, and LHWs involved in TS for POCT.

An electronic questionnaire, ‘Assessment of Point-of-Care Task-sharing Environment’ (APoCTe), was developed (S1 Text) and it collected semi-quantitative and qualitative data across four domains: policies and governance of TS for POC tests; LHW training and deployment; monitoring and sustainability; and costs or impacts of TS models. The tool was adapted to the Kobo Toolbox platform and piloted in English, French, and Portuguese-speaking countries (DRC, Botswana, and São Tomé and Príncipe).

Over 300 potential informants were contacted between November 2024 and January 2025 via standardized emails with a link to the APoCTe survey, followed by two reminders to boost response. The survey was administered online in English, French and Portuguese and it included closed and open-ended questions. Data were deposited in the Dryad repository [31]. Afterward, KII were conducted with selected national laboratory directors or POCT coordinators, based on availability and consent. Participants received study information and interview guides in advance. Three experienced researchers (ZN, KCL, and NF), trained in data collection tools, conducted the KII in the interviewees’ preferred language (French or English). Oral consent for participation and audio recording was obtained before interviews, which lasted about 30 minutes. KII explored the role of national laboratory departments in scaling POCT and task sharing with LHWs, with questions tailored from survey and document review findings. Interviews were transcribed, de-identified, and securely stored.

Participants were asked to provide recent versions of these documents: national health policies and strategies, national human resources for health strategies, vertical disease program strategies (HIV/AIDS, TB, malaria, NCDs), maternal and child health strategies, community health strategies, national essential diagnostic lists (NEDL), national laboratory strategies and policies, Covid19 preparedness plans, national task sharing policies, and POCT implementation policies or frameworks. An online search was also conducted on government and health ministry websites to locate these documents. Each document underwent full-text review using specific keywords related to POCT and task sharing. Data were extracted into a Microsoft Excel spreadsheet.

Country responses were cross-checked and validated across different respondents; and discrepancies reconciled using supporting documents and consultations with ASLM LabCoP teams. Findings were also compared with prior ASLM work, especially on NEDLs [32]. Final draft reports were shared with national laboratory leadership for verification.

Survey responses from the Kobo APoCTe tool were extracted into spreadsheets for analysis. Data from multiple respondents per country were compiled, summarized by theme, and compared with supporting documents. Descriptive statistics were used to summarize participant characteristics; categorical data are shown as frequencies (%) and medians with interquartile ranges for non-normal numerical data.

National policy and strategic documents were independently reviewed by researchers (ZN, MIA, KCL, HMS, RN) to extract information on POCT scale-up plans, TS with LHW, range of task-shiftable tests, user-training, supervision, and monitoring. French and Portuguese documents were reviewed by native speakers (KCL, RN, HMS and MIA) who then translated the findings to English. Findings were discussed and cross-verified in online meetings. Descriptive statistics summarized document characteristics, with categorical data presented in bar charts. Analysis focused on identifying gaps, opportunities, and examples of TS for POCT. A policy status dashboard was developed. The STROBE checklist [33] guided comprehensive reporting.

Qualitative findings were analyzed and reported following the COREQ checklist [34]. Audio and written KII transcripts were verbatim transcribed and translated into English by two independent translators then analyzed manually using thematic analysis. Key discussion points were highlighted and thematically grouped using framework analysis, guided by APoCTe questionnaire themes. Findings were discussed in online meetings with researchers (CO, MIA, FO, ZN).

### Study outcomes

1. Proportion of countries with national health policies, vertical disease program policies, or laboratory strategic plans that include TS for POC diagnostics with LHW.
2. Proportion of countries with costed implementation frameworks for scaling quality POCT access, including TS with LHW.
3. Proportion of countries with training, supervision, and quality monitoring frameworks for TS in POCT.
4. The qualitative descriptions of enablers and barriers for scaled up implementation of task shared POC testing with LHW.

## Results

### 1. Review of national policy and strategic guidance documents

A total of 217 national health policies, strategic documents, and implementation plans from 19 countries were reviewed, and 197 (91%) were deemed relevant, Table 1. Year of document validity ranged from 2009 to 2033 (median 2021–2025), with 79% published since 2020. Majority of the POC TS-related testing guidance reviewed, was part of national vertical disease programs (76/197, 39%), laboratory strategic plans (19/197, 10%), and health strategic plans (19/197, 10%). A full list is in S2 Text.

**Table 1:** Types of country documents deemed relevant and reviewed.

Document type	Number of documents (%)
National strategy for human resources for health (NSHRH)	5 (2%)
National health strategic plan (NHSP)	19 (10%)
National vertical disease strategic plans (HIV and or STI*, TB, NCD and malaria)	76 (39%)
National laboratory policy (NLP)**	7 (4%)
National laboratory strategic plan (NLSP)	19 (10%)
National essential diagnostics list (NEDL)	12 (6%)
National POC testing policy or implementation or certification guidance	11 (5%)
National community health (or worker) strategy**	7 (4%)
National Covid19 preparedness response plan	13 (6%)
Maternal, newborn and child health care strategic plan	13 (6%)
National task shifting and task sharing policy	1 (1%)
Other (*national laboratory operational plan*, *national standards for medical laboratories, national universal health coverage plan, national multidisease testing framework, supplemental strategic documents*)	14 (7%)

Key: * STI strategic plans for majority of the countries were embedded within the HIV/AIDS strategic plans; ** a substantial number of respondents mentioned that this document is embedded primarily within broader ‘national health policy or strategic plan’ document.

i. National health strategic plans (NHSP)

**Fig 1:**
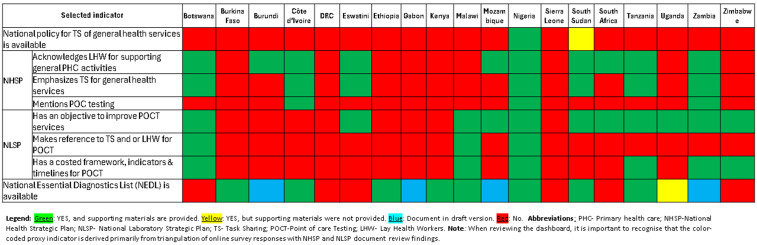
Dashboard for national health strategic plans and national laboratory strategic plans, regarding task shared POCT with LHW.

Over half of country NHSPs (10/19; 53%) recognize LHWs, especially community health workers, as vital for the expansion of PHC services, but fewer (7/19; 37%) mention TS. Zambia and Nigeria specifically highlight strengthening TS for POCT, Fig 1. Tanzania’s NHSP (2015–2020) calls for reviewing laws to enable TS, emphasizes competency training, and highlights the role of academic institutions and professional bodies in formalizing LHWs for TS.

ii. National strategy for human resources for health (NSHRH)

Only 5 of 19 (26%) countries had accessible NSHRH documents (Burkina Faso, Burundi, Côte d’Ivoire, DRC, South Africa), and all listed multidisciplinary HCWs (doctors, nurses, pharmacists) available/needed for adequate service delivery. However, only South Africa’s document included estimated available LHW numbers (CHWs, lay counsellors) and modelled human resource shortfalls for improving PHC utilization.

iii. National laboratory policies (NLP)

Among the 7 received NLP, only Nigeria’s NLP (2023) emphasized the importance of POCT, but also highlighted limited guidance on its utilization. It also highlighted the need for training, quality and supervision of POC testers in non-laboratory settings. The Malawi NLP (2023–2030) highlighted laboratory technician shortages and mentioned TS to LHW, however, it also stated that LHW have no formal technical training and that the Medical Council of Malawi did not recognize or register them.

#### Dashboard for national health strategic plans (NHSP) and national laboratory strategic plans (NLSP) regarding task shared POCT.

**Fig 2:**
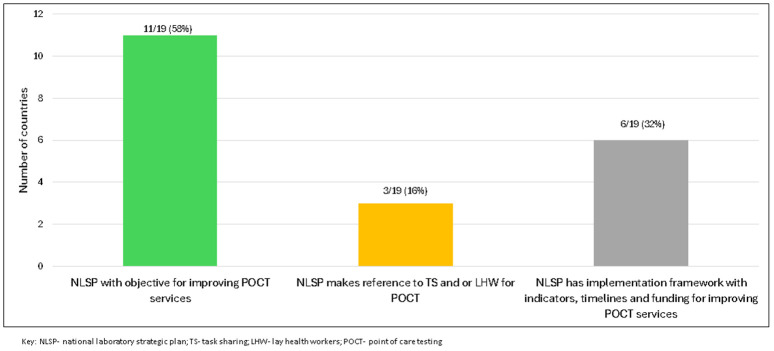
Inclusion of task sharing for point-of-care testing with LHW in national laboratory strategic plans.

Fig 2 provides a snapshot of findings triangulated from the online survey and review of NHSP and NLSP documents. The dashboard may not speak to the level of implementation of LHW-led TS for POCT in healthcare facilities across different countries.

iv. National laboratory strategic plan (NLSP)

Of the 19 countries submitting NLSP, 11 (58%) included plans to improve access and quality of POCT (for example; tester and site certification, POCT guidelines, external quality assessment) in facilities with or without laboratories, Fig 1 and Fig 2. However, only 3 (Botswana, Malawi, Nigeria) mentioned TS for POCT with LHW, Fig 2.

Among countries planning to improve POCT access, 6/19 (32%) NLSPs (Botswana, Malawi, Nigeria, Tanzania, Zambia, Zimbabwe) included implementation frameworks with timelines and indicators whilst costing plans were noted for all except Botswana and Zimbabwe, Fig 2. Costing plans covered guideline and training manual development (Malawi, Nigeria, Zambia), site certification and tester training (Nigeria), quality assurance and supervision (Nigeria, Tanzania, Zambia), and POCT evaluation (Nigeria). The need for donor support to fund improving POCT services was explicitly mentioned in four NLSPs (Malawi, Nigeria, Tanzania, Zambia). Nigeria’s NLSP provided detailed plans with smart-indicators, timelines, and funding plans for POCT in non-laboratory settings. Additional elements related to strengthening POCT services mentioned in NLSP included support for POCT validation agencies, sensitizing health facilities and regulatory bodies on the NEDL, and promoting local in-vitro diagnostic production (all Nigeria).

All southern African countries (7/7; 100%) included POCT scale-up objectives in their NLSPs, followed by east Africa (3/6; 50%), west Africa (1/4; 25%), with central Africa least likely (0/2).

v. Vertical disease program strategic plans

**Fig 3:**
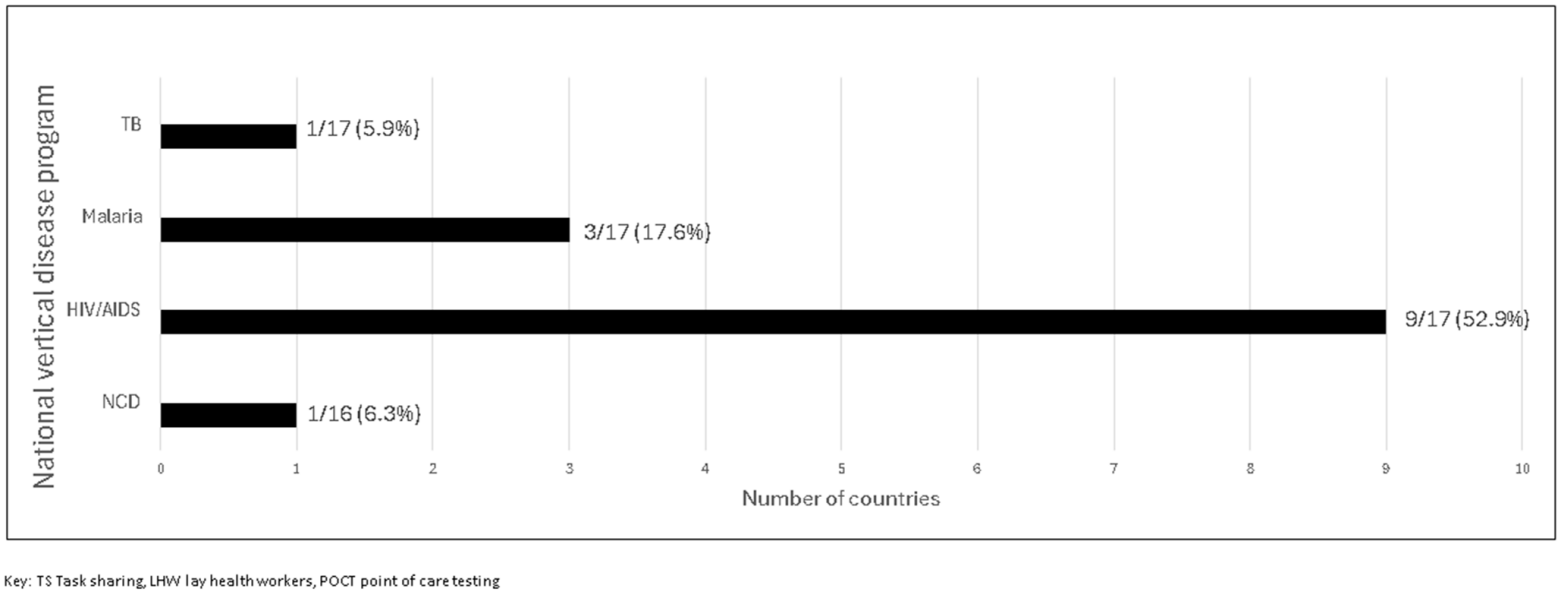
Inclusion of task sharing and or LHW for POCT services in national vertical disease strategic plans.

Of 17 national HIV/AIDS strategic plans received, 9 (53%) mentioned TS for POCT with lay cadres (Burundi, Côte d’Ivoire, Malawi, Mozambique, Nigeria, Sierra Leone, South Sudan, Tanzania, Zimbabwe), Fig 3. Malawi’s HIV/AIDS strategic plan (2017–2022) noted human resource shortages for HIV testing services and emphasized deploying LHW (health diagnostic assistants) for POCT. Zimbabwe’s HIV/AIDS and STI strategic plan (2021–2025) detailed a framework for improving POCT, including training, supervision, enrolment of POC sites into proficiency testing programs, and mentions TS to LHW for HIV, syphilis, hepatitis, and molecular tests, plus maintaining an active tester inventory shared with reference laboratory. Advanced HIV disease POCT (CD4, LAM, CrAg) was scarcely mentioned in the national HIV/AIDS strategic plans, except in Zimbabwe’s 2022 operational manual, which mentions that AHD POCT should also be performed in the community by trained cadres (nurses, primary counsellors and microscopists).

Among NCD national plans received, only Nigeria’s NCD plan (2019–2025) mentioned TS for POCT with LHW and has a priority action to expand the TS policy to build capacity of health workers at all levels of care to improve diagnosis and management of NCDs. Eswatini’s NCD strategic plan emphasizes TS for the screening of NCDs with CHW however, it does not mention POCT.

Among 17 TB national strategic plans, only Nigeria’s (2021 – 2026) emphasizes TS and the need to strengthen TB diagnosis at all health levels. It also mentions engaging professional bodies and academic institutions to support training and TS and has a log framework with indicators timelines and responsibilities for all these activities. It also emphasizes the need to assess the impact of TS in improving child TB notification and management (integration).

Of the 17 national malaria strategic plans received, only Burundi, Malawi, and Zambia mention LHW (CHWs, community health assistants) in the diagnosis and or management of malaria, Fig 3. However, Malawi’s malaria strategic plan states that a national task shifting policy was not developed during 2017–2022 due to conflicts with the medical council’s legal framework.

vi. National POC testing implementation guidance

Eight countries (Côte d’Ivoire, Kenya, Malawi, South Africa, South Sudan, Tanzania, Zambia, Zimbabwe) submitted national POCT policies or implementation guidance focusing on general implementation (training, quality, supervision, certification). Three (Côte d’Ivoire, South Sudan, Zambia) specifically focused on HIV testing, while others covered broader POCT including near-POC molecular, biochemistry, and immunoassays. For Zimbabwe, the document mentions that LHW to conduct POCT must be registered with the Medical Laboratory and Clinical Scientist Council (MLCSCZ) and or other council. The testers are required to have a valid competence record from MLCSCZ. For Tanzania and Zambia, their POCT certification frameworks do not require professional registration but mandate training, certification, and periodic refresher courses, with oversight by the Health Laboratory Practitioners Council (HLPC). Certificates last two years (Zambia) with re-certification based on competency. Those who have poor performance in re-certification will undergo mentorship and continuous education.

South Africa’s draft POCT policy (2020) assigns lateral flow assay oversight to the National Department of Health (NDoH) and device-based POCT to National Health Laboratory Services (NHLS). However, NHLS will support NDoH with provision of quality assurance and evaluation of new POC tests. South Africa also has a draft POCT implementation plan (2025) which seeks to guide POCT rollout. However, these two draft documents do not emphasise LHW or task sharing. Operators of device based POC and near POC should be registered with the health professions council of South Africa (HPCSA).

vii. National essential diagnostic lists (NEDLs)

Eight countries (Burkina Faso, Côte d’Ivoire, Ethiopia, Kenya, Malawi, Nigeria, South Sudan, Tanzania) had published NEDLs, whilst four (Burundi, Gabon, Mozambique and Zambia) had draft versions. These NEDLs list the range of POC tests that can be conducted in laboratory and non-laboratory settings including in community settings, however, none of them highlight which POC tests can be task shared with LHW (after adequate user training). Nevertheless, Malawi’s NEDL outlines the minimum required human resources for conducting POC tests and states that trained lay personnel, such as microscopists, are qualified to perform various POC and near-POC tests.

viii. Community health worker strategic (CHW) plans

Of the 7-community health strategic plans, all mention that CHW can conduct HIV testing services, however, Botswana’s CHW plan emphasizes other POC tests that CHW can conduct (HIV, glucometers, urine strips).

ix. Covid19 preparedness response plans

None of the 13 national Covid19 preparedness response plans emphasized TS, LHW or POCT.

x. National task sharing strategic plans or policy

Only one national task sharing policy for essential health services was accessed (Nigeria, 2014) and it had a framework for empowering LHWs to support the scaling up of access to essential health services in maternal and newborn, malaria as well as for HIV and TB services, especially at PHC and community levels. TS for POCT is emphasised for HIV, malaria, and TB services. The document also recommended establishment of training programs with certification, registration of trained LHW with regulatory agencies, supervision and mentorship services as well as fair remuneration.

### 2. Online survey participants and their responses

#### Characteristics of online survey study participants.

Seventy-five key stakeholders from 17 African countries completed the online survey; 4 (5%) declined. Respondents included 4 (5%) from Botswana, 3 (4%) Burkina Faso, 5 (7%) Burundi, 3 (4%) DRC, 4 (5%) Côte d’Ivoire, 2 (3%) Eswatini, 3 (4%) Gabon, 4 (5%) Kenya, 11 (15%) Malawi, 10 (13%) Mozambique, 5 (7%) Nigeria, 3 (4%) Sierra Leone, 4 (5%) South Sudan, 1 (2%) Tanzania, 4 (5%) Uganda, 4 (5%) Zambia, and 5 (7%) Zimbabwe. Most (57%) were from national ministries of health, followed by implementation partners (28%). Half (51%) had held their current work-position for at least four years, Table 2.

**Table 2:** Characteristics of study participants for the online survey.

Variable	Proportion, n (%)
**Total**	75
**Sex**	
Female	25 (33.3)
Male	50 (66.7)
**Age range (years)**	
20-30	2 (2.7)
31-40	21 (28.0)
41-50	22 (29.3)
> 50	30 (40.0)
**Employer (*and or role*)**	
National ministry of health (policy)	14 (18.7)
National ministry of health (disease program implementation)	16 (21.3)
Public laboratory	10 (13.3)
Private laboratory	5 (6.7)
Professional laboratory council/association	1 (1.3)
Medical council/association	2 (2.7)
Implementing supporting partner	21 (28.0)
Lay cadres involved in POCT services	2 (2.7)
Other	4 (5.3)
**Work position**	
Director/deputy director	13 (17.3)
Manager (laboratory, program)	26 (34.7)
Laboratory scientist/technician/advisor	24 (32.0)
Other	12 (16.0)
**African region representativeness of countries included ∞**	
North Africa	0
Central Africa **β**	2 (11.8)
East Africa **Ω**	5 (29.4)
Southern Africa **$**	6 (35.3)
Western Africa **£**	4 (23.5)

**Key**: **∞** Categorization as per African Union regions [35].

**β** DRC, Gabon; **Ω** Burundi, Kenya, Tanzania, Uganda, South Sudan; **$** Botswana, Eswatini, Malawi, Mozambique, Zimbabwe, Zambia; **£** Burkina Faso, Côte d’Ivoire, Nigeria, Sierra Leone.

#### Online survey responses.

a) Training and deployment of LHW for task shared POC testing

All respondents reported that LHW conduct POCT across public and private health facilities as well as in community settings. Tests included CD4 cell counts, blood sugar, rapid tests (HIV, syphilis, hepatitis, malaria, pregnancy, urine TB LAM, CrAg), and near-POC molecular tests like GeneXpert for HIV early infant diagnosis and viral load. LHW cadres conducting POCT included phlebotomists, counsellors, healthcare auxiliaries, peer workers, community health workers, health care assistants, care givers, diagnostic health assistants, health extension workers and malaria agents. Implementation partners were mentioned to mainly provide remuneration (85%), sometimes alongside governments. Few respondents mentioned that in some instances, LHW are volunteers and sometimes not salaried or given an occasional stipend or incentive.

Over 65% noted rapid HIV test training as the most established program, and criteria for training was either being an HIV counsellor or those with high school education; with certification issued upon completion. Partners were mentioned to largely support the trainings. In Botswana LHW undergo 8-week ministry of health approved HIV testing and counselling course with a partner organization; Eswatini includes a 2-month facility attachment. For Botswana a respondent mentioned that there is a database of all LHW trained in HIV RHT services including details of health facilities that have human resource shortages. In Zimbabwe, trained nurses or laboratory technicians cascade the training within vertical programs.

Participants highlighted that training for other POCTs (hepatitis, syphilis, malaria, AHD) varies widely, mainly managed by vertical disease-specific programs and national laboratories, but there is no standardized curriculum.

Nearly all respondents reported that national laboratory service mapping, aided by partners, was conducted and focused on instruments and staff but excluded PHC testing needs like LHW availability and POCT training.

b) Quality assurance in LHW-led POCT and governance

Seventy-six percent (47/62) of respondents reported a proficiency testing (PT) program where national laboratory sends blinded samples to LHW for testing, mostly for HIV (66%). PT panels are sent biannually in countries like South Sudan, Kenya, and Mozambique; Eswatini is expanding to syphilis and hepatitis. Botswana also has a laboratory master trainer program providing supervision and mentorship to LHW.

Most respondents (87%; 60/69) said LHW are not registered with a professional body but are overseen by vertical disease programs or nearby laboratories. In Zambia, HIV counsellors conducting POCT are registered with the Zambia counselling council and recertified every two years.

### 3. Key informant interviews

Of seven identified national laboratory directors, six (Côte d’Ivoire, Ethiopia, Malawi, Nigeria, Kenya and South Africa) participated in the KII. The KII qualitative data was synthesized into two primary themes which include: (1) facilitators and (2) barriers for the scaled-up implementation of task shared POC testing.

#### Theme 1: Facilitators to the scaled-up implementation of tasked shared POCT with LHW.

All respondents agreed on the value of TS for POCT and on the implementation gaps but expressed varied opinions on enablers for scaling up TS as illustrated by some quotes below.

One respondent said “…*to improve efficiencies and governance in laboratory services including institutionalizing TS for multidisease POCT, it is crucial to have the laboratory department have its own directorate with its own resources directly under the authority of the ministry of health as opposed to falling under multiple entities or institutions and having to be dependent on resources from vertical disease-specific programs, to lead basic scale-up of POCT services (like training, supervision)*. *Having a standalone directorate for the laboratory sector with its own dedicated funding could enhance operational autonomy, improve resource allocation, increase efficiency and strengthen governance..*.”

Another responded mentioned that: “…*there is a need to agree on a minimum package of POC tests doable by lay cadres*…”. The respondent further added that: “….*to harmonize POC tester user training for the many POC tests, it may be more feasible to utilize the medical training centres that primarily train lay cadres for their primary roles..*.”

#### Theme 2: Barriers to the scaled-up implementation of task shared POCT with LHW.

Respondents highlighted several challenges that hindered the scaled-up rollout of task shifting for POCT with LHW.

A respondent mentioned that “…*even though TS has been determined to be priority, the fiscal arrangements required to support its implementation, as well as coordination between laboratory departments, vertical disease programs, and the professional associations, has been ineffective...*”

Another respondent mentioned that: “…*the term preferred by most health care workers, especially from laboratory services, is task sharing and not shifting the work, as we are just sharing with them. And this issue of task shifting has prior resulted in court battles.….in fact, the laboratory directorate finds it difficult to mention TS for POCT with LHW in its strategic plans because of these court rulings and ongoing pressure against task sharing…*.”

Another respondent highlighted that “…*for many countries, TS for POCT could have been easier and less complex to implement in vertical programs as opposed TS for multi-disease POCT, however, multi-disease POCT could be more sustainable and more impactful*…”

## Discussion

This study reveals progress in the implementation of TS for POCT in African countries; however, this is mostly in fragmented disease-specific approaches and numerous national health policies and strategic documents do not emphasize TS for POCT with LHW. For sustainable and equitable POCT, coherent policy and implementation reforms are urgently needed to institutionalize TS for POCT to LHW, especially in an era of declining resources. National laboratory leadership must play a central role in the scaling up of TS for multi-disease POCT.

Within their over-arching national health strategic plans, over half of the countries (53%) acknowledged the importance of LHWs in improving primary healthcare, however, fewer (37%) emphasized TS with LHWs to scale up POCT. Compounding this inefficiency is the subsequent lack of reference to integrated task shared POCT in most multi-country vertical disease program strategic plans (79%) and national laboratory strategies (84%). While some countries permit LHWs to perform TS for POCT, the absence of formal supportive policies can hinder decentralization efforts. Aligning national health strategic plans, vertical disease programs, and national laboratory strategic plans is crucial to support integrated, sustainable, and effective TS for POCT.

While most national laboratory strategic plans (11/19; 58%) emphasize improving POCT access and quality, nearly three-quarters lack indicators to measure scale-up progress and hold implementers accountable. Cost projections and funding sources for improving POCT services were largely missing in most country NLSP, with only 26% providing such details. This lack of financial planning undermines feasibility and implementation of proposed activities, and this risks NLSP being aspirational rather than actionable. As countries develop their five-year national laboratory plans [36], they should prioritize operationalizing task-shared POCT services, recognizing that laboratory services hold ultimate oversight responsibility of testing services, especially as the range of POC tests is expanding.

It is encouraging that many countries have national implementation or certification guidance for POCT, but in half, these apply only to HIV testing. This guidance should expand to include other POC tests as well as comprehensive supervision and monitoring strategies, especially as adoption of LHW-led task sharing increases testing devices and operators. In some countries like Zimbabwe, the national implementation and certification framework prerequisites that testers must be registered with professional laboratory or health councils, and this may exclude LHW involvement in task shared POCT, as most survey respondents reported no council exists for LHW registration.

Adopting a national essential diagnostics list (NEDL), which includes in-vitro diagnostics for use outside laboratories, is a practical and impactful step for advancing POCT services and should be prioritized [10,32]. Nevertheless, all the currently published NEDLs from 8 countries do not specify required tester skills (professional or LHW) nor clarify which POC tests can be task-shared with LHWs. Including this information in NEDLs could help national programs develop clear policies on the range of acceptable POC tests for less-trained health workers.

Although text review of national policy documents did not find major mention TS of POC tests with LHWs, all online survey respondents reported that LHWs conduct POCT in their countries. These activities are mostly confined to vertical, disease-specific programs and are not integrated into broader health protocols. This approach limits LHWs to narrow testing roles and restricts comprehensive access to POC tests. Most funding for TS in POCT was mentioned to come from international partners focused on vertical disease programs, which, while improving access to disease-specific testing, further reinforced this fragmented approach. However, given abrupt funding terminations to implementation partner activities [30], TS for POCT could fall off the global health agenda unless national programs explore adaptations towards sustainable strategies for TS for POCT.

Harmonized training for LHWs in POCT currently exists mainly for HIV RDT services. Building on these models, national laboratory departments, in collaboration with laboratory councils or training institutions, should standardize training for other POC tests as outlined by the EDL [10] or NEDL. Training could include pre-service, in-service, and remote learning options. LHW training and mentorship support should also include management of confidentiality, privacy and stigma associated with testing as this is critical to the acceptability and success of decentralised testing approaches. To enhance workforce efficiency, education programs must be updated to reflect evolving LHW roles and support career development. Training should also cover CHWs, together with clarity on the minimum POCT package they can use during community health activities. Assessments are needed to evaluate workforce availability and deploy trained LHWs to areas with the greatest shortages, especially at PHC levels. In many areas, it is unclear if enough LHWs exist to share tasks with. Without surplus capacity, TS could have the unintended consequence of reducing time for other vital activities. LHW should be part of broader national health workforce policies and planning supported by structured training programs and adequate remuneration as well as regulatory frameworks.

### Recommendations

Setting priorities within national health services is a process, driven not just by evidence of utility of TS, but also by the power of policy actors, prevailing ideas and the emergence of windows of opportunity [37,38]. To minimize TS for POCT from becoming a meagre program, countries should establish a POCT task force or working group constituted of representatives from different vertical disease programs, laboratory leadership and professional councils, implementation supporting partners, civil society organization (CSO) and or community representatives. The task force will provide oversight for the institutionalization of TS for POCT. Institutionalization of TS for POCT refers to the formal integration of TS for POCT with LHW across disease programs into the broader health system, especially at PHCs. This involves national health policy and strategy documents emphasising the role of LHW for integrated multi-disease TS for POCT, ensuring they are formally trained, supervised, and compensated, including integrating their roles within existing health structures. In addition, this will involve ensuring availability and access to diagnostic supplies in PHCs necessary for LHW to conduct multi-disease POCT. Institutionalization could improve the effectiveness and sustainability of LHW-led POCT and subsequently increase equitable access to POCT in public health.

**Fig 4:**
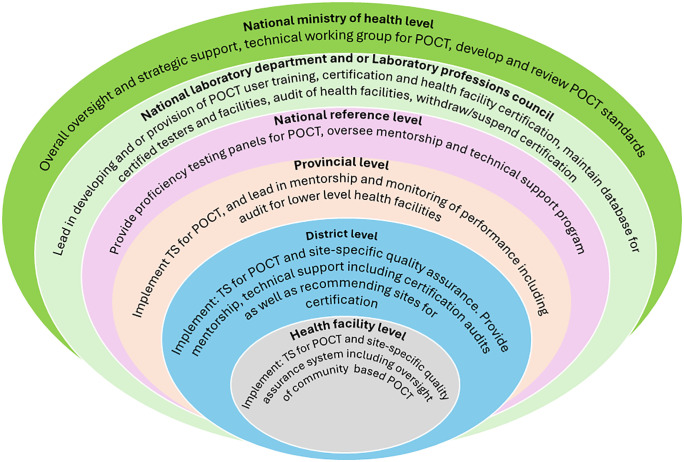
Governance structure for the point-of-care testing program. Adapted from the WHO handbook of improving the quality of HIV related POC testing [39].

Table 3 and Fig 4 below, show a visual layout of other key enablers and governance through which integrated multi-disease TS for POCT is anticipated to be institutionalized in national health systems through a structured and comprehensive approach. These enablers are derived from study findings and literature review.

**Table 3:** Enablers towards institutionalization of TS for POC tests with LHW.

Policy and strategic guidance documents	Interventions	Expected outcomes and or long-term impact
What’s needed: • NHSP should emphasize importance of scaling up POCT services towards realization of health initiatives like UHC and acknowledge the utility of TS particularly with LHW. • National ministry or department of health should lead in the development of harmonised national norms and standards to promote general TS in health services. • The national human resource for health policy should recognize and emphasize the importance of LHW as essential for supporting efforts in public health service and provide clarity for their employment. • NLSP should have a detailed cost projection for scale-up of POCT, including monitoring plans. NSLP should also acknowledge the role of TS for POCT with LHW and provide guidance on how to operationalize TS for POCT. • Vertical disease-specific program strategic documents should emphasize the integrated multi-disease role of LHW for POCT and these documents should align with NLSP about POCT including TS. • National POCT certification and implementation guidelines should outline strategies for LHW training, supervision and monitoring for multi-disease POCT. • NEDL and or national POCT guidelines should stipulate the range of task shiftable POC tests.	What’s needed • For dissemination of new or revised national health policy and strategic documents, ministry of health should engage a wide range of stakeholders and use effective communication strategies for wide reach. • National laboratory services together with vertical disease programs should explore testing needs at PHC facilities and availability of LHW including their roles. • National laboratory services, laboratory professions councils and or key national training institutions should lead the harmonization of POC tester training with certification and or continuous professional development programs. • Ministry of health to ensure salaried deployment of LHW for POCT. • National laboratory should lead the monitoring, supervision and quality assurance program for POCT. • CSO should lead advocacy for the development of national TS roadmaps and implementation.	• Empowered LHW trained and capable of conducing multi-disease POCT. • Institutionalization of LHW and TS for POCT. • Increased availability and use of POC at PHCs. • Increased POCT rates in health facilities.

Assumptions and enablers: The national health sector procurement and supply chain will assure availability of POC tests in PHCs. There is availability of LHW, particularly in PHCs.

Key: NHSP- National Health Strategic Plan; NLSP- National Laboratory Strategic Plan; CSO- Civil Society Organizations.

Countries should promptly integrate proven interventions into public health strategies, especially as the global health financing landscape is undergoing rapid changes which also impact diagnostics and health services [30].

This study’s strengths include a large, diverse sample of respondents from 19 African countries, triangulating data from surveys, key informant interviews, and policy reviews, with findings verified by country teams for robustness. However, the purposive selection of countries enrolled in ASLM’s LabCop may limit continental representativeness. Of 22 eligible countries, three were excluded due to non-response. The study findings are mainly based on documents that were accessible, and we acknowledge the possibility that findings could have been different, if all national documents had been available. Although we aimed to review the most currently available policies and strategic documents, some were not accessible, some were out of date and or in the process of being updated. As this study is focused on collecting information pertaining to national testing policies, it is possible that some information on LHW testing may not have been specified in these but rather included in other provisions that allow LHW to perform TS for POCT. However, all efforts were made to explore availability of such information from all sources. Documents in French and Portuguese were translated by native speakers with cross-checks to minimize errors, though some nuance may have been lost. Funders of national testing programs as well as the voices of community members were not included in the study, potentially overlooking important insights. Finally, the low participation of LHWs in the survey, may limit the representativeness of their perspectives.

Future studies could explore the legal frameworks within professional councils (laboratory and or medical) which could be causing policy uncertainty and bottlenecking task shared POCT initiatives. Other studies could explore the availability and accessibility of essential diagnostic tests in health facilities, particularly in decentralized PHC.

## Conclusion

TS of POCT to LHWs is common in many countries, primarily within vertical, disease-specific programs and with significant donor support. Transitioning from fragmented, disease-specific approaches to integrated, health system-wide task-sharing models is essential for building more sustainable, equitable testing systems. Coherent policy and implementation reforms are urgently needed to institutionalize task-sharing for POCT to lay providers in an era of declining resources and increasing diagnostic needs. National laboratory leadership must play a central role in the adoption and implementation of simplified, multi-disease training and quality assurance frameworks. Recommendations in this report should guide national policy and strategic health planning.

## Supporting information

S1 Text Semi-structured interview questionnaire (Assessment of Point-of-Care Task-sharing Environment’ (APoCTe)) used for key informant interviews. (DOCX)

S2 Text List of national documents reviewed. (DOCX)
