# Supplementary material for: Task sharing for point-of-care testing: Review of national health policies and implementation landscape in 19 African countries
Source: PLOS Glob Public Health. 2025 Dec 29;5(12):e0005485. doi: 10.1371/journal.pgph.0005485 (PMC12747379; doi:10.1371/journal.pgph.0005485)
Supplement: S1 Text — (DOCX) [file pgph.0005485.s001.docx]

S1 Text: Semi-structured interview questionnaire (Assessment of Point-of-Care Task-sharing Environment’ (APoCTe)) used for key informant interviews.

Unique study ID..……………………………Age range………….Gender…………

| Work position…………………………………. | | | How long have you held this position for? ……….......................(years) | | | |
| --- | --- | --- | --- | --- | --- | --- |
| Briefly explain your work role/s…………………………………………………………………………………………. | | | | | | |
| Employer: Ministry of health **[ ]**, partner/NGO **[ ]**, Lab council leadership **[ ]**, Lab operational **[ ]**, Disease program operational **[ ],** Other **[ ]** | | | | | | |
| Date of response (*dd/mm/yy*)………Time of interview (HH:mm)…………. Country of respondent…........................................ | | | | | | |
| **SECTION 1: Policy and laws** | | | | | | |
|  | | **Question** | | **Explanation** | | **Answer** |
| 1.0 | | Is there an adapted national Essential Diagnostics List (NEDL) as per WHO EDL guidance?  Please share a copy (if ‘Yes’). If ‘NO’, are there plans to adapt the NEDL? | | This question seeks to explore presence of the NEDL (even if draft version), or for plans to consider its implementation. In the absence of NEDL there may be a tier specific testing strategy, it may be worth asking also for these alternatives | | This can be a ‘Yes’, ‘No’ answer. But needs to be elaborated further |
| 2.0 | | Has there been any mapping exercise conducted to explore the availability of laboratory-trained workforce in facilities with/without laboratories? | | This question seeks to gauge if there is any in-depth understanding of the densities of lab-trained human resource availability in health facilities with/without laboratories. Together with optimum required targets | | This can be a ‘Yes’, ‘No’ answer. But needs to be elaborated further |
| 3.0 | | Has there been any mapping conducted to explore areas/sites with potential demand for deployment of LHW for POC testing? Please elaborate more | | This question seeks to scope-out for activities to improve POC testing rates and/ support task shifting for POC testing, especially in areas with high demand | | This is an open ended question |
| 4.0 | | Is there a dedicated national policy (and or strategy document) that supports LHW to conduct specimen collection (phlebotomy)? | | This question concerns all different sample collection procedures: dried blood spots, finger prick, and venepuncture. | | [Yes], [No], [Other]  *(tick one that applies)* |
|  |  | If ‘YES’ please attach a copy of this policy or strategy document. | | | |  |
| 5.0 | | Is there a dedicated national policy (and or strategy document) or human resource document that supports task shifting/sharing of POC tests to LHW? | | This question concerns availability of any national document (even if in draft format) that supports LHW conducting POC tests in health facilities | | [Yes], [No], [Other]  *(tick one that applies)* |
|  |  | If ‘YES’, please give details about the task shifting/sharing policy and or strategy:   - Has it been broken down into job aids or SOPs When was it developed/enacted? - Has it been shared to all stakeholders (PHCs, lab councils, medical council etc.)? - Is it part of national HR policy? | | | | This is an open ended question |
| 6.0 | | Is there a programmatic policy (and or strategy document) that supports task shifting/sharing of POC tests to LHW? | | Task shifting policy or guideline/s might be contained in various documents; for instance, they could be contained in the NEDL or in specific national disease testing strategies (HIV, TB, maternal health, etc) or in the minimal testing packages manual or in the manual for the user of the lab networks | | This can be a ‘Yes’, ‘No’ answer. But needs to be elaborated further |
|  |  | If YES’, please give details about the programmatic policy document:   - Where is it contained (in the minimal testing packages, or NEDL or manual for the user of the lab networks or in disease specific strategies (HIV, TB, maternal health, etc)? Has it been shared to all stakeholders (PHCs, lab councils, medical council etc.)? - Please share a copy | | | | These are open ended questions |
| 7.0 | | Is there a dedicated institutional policy (and or strategy document) that supports task shifting/sharing of POC tests to LHW? | | Similarly, task shifting policy or strategy documents could be contained in institutional documents | | ‘Yes’, ‘No’ answer. But needs to be elaborated further |
|  |  | If ‘YES’, please give details about the task shifting/sharing policy and or strategy | | | |  |
| 8.0 | | Are there any laws/acts that explicitly prohibit LHW from performing POC tests? | | This concerns known laws/acts or overt ‘culture/practise’ that criminalizes/bans LHW from conducting POC tests. | | [Yes], [No], [Other]  (tick one that applies) |
|  |  | If ‘YES’, please attach a copy for such law/act or policy or describe the overt ‘culture/practise’. | | | |  |
| **SECTION 2: Training, deployment and roles** | | | | | | |
|  | | **Question** | | **Explanation** | | **Answer** |
| 1.0 | | Generally, is there any POC testing conducted by LHW in your country? | | The questions ask about task shifted POC testing in the community or facilities. | | [Yes], [No], [Other]  (tick one that applies) |
|  |  | If ‘YES’, is this LHW led testing happening in health facilities or community outreach activities or both? | |  |  |  |
| 2.0 | | What is the job profile/name of these LHW who conduct POC testing? | | Depending on their roles, LHW are sometimes referred to as HDA, CHW, lay counsellors, phlebotomists, health surveillance assistants, health facility navigators, among other names. | | This is an open ended question |
| 3.0 | | Who pays their remuneration/salaries? | | Ministry of health, partners/NGOs, un-salaried | | This is an open ended question |
| 4.0 | | Which POC tests do they conduct? | | HIV, Malaria, CD4, Syphilis, Glucometers, etc | | Open ended question |
| 5.0 | | Is there any formal training services for LHW to conduct POC testing? | | This concerns training through private or national services program | | ‘Yes’, ‘No’ answer. But elaborated further |
|  |  | - What is the entry criteria? What does the training cover? - Who conducts the training (organization, school/college)? - Is there certification post training? - How many LHW have been trained so far? Do trained LWH join any professional body? - Are there clear carrier growth paths for trained LHW? | | | | These are open ended questions |
| **SECTION 3: Supervision, monitoring and sustainability** | | | | | | |
|  | **Question** | | | **Explanation** | | **Answer** |
| 1.0 | Which entity/body governs the LHW? | | | This could be the laboratory council, or medical council or others | |  |
| 2.0 | Are LHW registered to any council for practise? Is there an association to advance and protect LHW profession? | | | | | These are open ended questions |
| 3.0 | What schemes or systems exists for monitoring the quality of POC testing services conducted by LHWs in PHCs and other facilities? | | | This questions concerns availability of quality control mechanisms in place at health facilities to monitor quality of POC testing by LHW. Or it could be ongoing supportive supervision by hub laboratories to PHCs/sites with LHW led POC testing. | | This is an open ended question |
| **SECTION 4: Costing and impact of task shared/shifted POC testing services** | | | | | | |
|  | **Question** | | | | **Explanation** | **Answer** |
| 1.0 | What could be the key cost drivers for implementing task shared services for POC testing? Are these elements costed and or covered by national budgets? | | | | This could be training needs, salaries, etc. This is a double question for the costs and national budgeting. | These are open ended questions |
| 2.0 | What is the guestimate cost to deploy the LHW responsible for POCT task shared/shifted testing services, in a year (salaries, support framework, etc)? | | | | This can be estimated from the probable total number of deployed LHW for POC testing and their annual salary needs | These are open ended questions |
| 3.0 | Has there been any exercise to explore if LHW-led task shifted POC testing in PHC facilities (without laboratories), is inferior to standard of care testing (without task shifting)? | | | | This question is not limited to comparison at PHC, but can also include community and or outreach | ‘Yes’, ‘No’ answer. But needs to be elaborated further |
| 4.0 | In your opinion, how can LHW best complement lab-based testing. In your opinion, what are the risks posed by LWH? | | | |  | Open ended question |
