# Supplementary material for: Task sharing for point-of-care testing: Review of national health policies and implementation landscape in 19 African countries
Source: PLOS Glob Public Health. 2025 Dec 29;5(12):e0005485. doi: 10.1371/journal.pgph.0005485 (PMC12747379; doi:10.1371/journal.pgph.0005485)
Supplement: S2 Text — (DOCX) [file pgph.0005485.s002.docx]

S2 Text. List of national documents reviewed

| **Country** | **Document/s reviewed, year** |
| --- | --- |
| Botswana | National non-communicable disease strategic plan 2018-2023  National health sector strategic plan 2010-2020  National laboratory services strategic plan 2024 – 2028  National guidelines on health service integration (*reproductive health, newborn child and adolescent health + nutrition, non-communicable diseases and other services*) 2021  National guideline for implementation of integrated community-based health services 2020 |
| Burkina Faso | National list of essential in vitro diagnostic tests by level of care 2023  National strategic plan to fight malaria 2021-2025  National health sector policy 2018 - 2027  National health strategic plan 2021-2030  National health development plan 2021 - 2030  National human resource for health development plan 2013 - 2020  National strategic framework to fight HIV/AIDS and sexually transmitted infections 2021-2025  National HIV/AIDS strategic plan 2021 - 2025  National laboratory strategic plan 2024 - 2026  National TB strategic plan 2024 – 2026  Supplement to the national TB strategic plan 2024 - 2026  National strategic plan for non-communicable diseases 2024 – 2028  National multisectoral strategic plan to fight non communicable diseases 2019 - 2021  National Covid19 response strategy-diabetes 2020  National laboratory policy 2007  National community health strategy 2024 - 2028  National family planning SP 2021 – 2025  Revised national Covid19 outbreak preparedness and response plan 2020  National integrated strategic plan for reproductive maternal, neonatal child, adolescent youth and elderly health 2017 - 2020 |
| Burundi | National malaria strategic plan 2021 - 2027  National human resource for health development plan 2014  National strategic plan to fight TB 2023 - 2027  National non-communicable disease strategic plan 2019 - 2023  National Integrated strategic plan for HIV/AIDS, sexually transmitted infections and viral hepatitis 2023 - 2027  National health sector policy 2016 – 2025  Norms and standards of medical biology laboratories  National health sector strategy 2021 – 2027 (*aligned with Burundi’s national development plan*)  National medical biology laboratory strategic plan 2021 - 2025  National multisectoral strategic plan for the control of non-communicable diseases 2019 – 2023  National strategic plan for reproductive maternal, neonatal child and adolescent health 2017 2020  National laboratory policy 2014  National STI framework 2019 - 2023  National response plan to the Covid19 pandemic 2021 |
| Côte d’Ivoire | National strategic plan to fight HIV/AIDS and sexually transmitted infections 2021-2026  National human resource for health development plan 2009 – 2013  National TB strategic plan 2021 -2025  National non-communicable disease strategic plan 2015-2019  National strategic plan to fight malaria 2021-2025 (extended to 2026)  National essential diagnostic lists 2024  National health development plan 2021 - 2025  National strategic plan for biomedical laboratories 2023 – 2025  HIV POC testing policy, standards, and procedures 2019  National essential diagnostics list 2024  National newborn action plan 2018 - 2020 |
| DRC | National HIV/AIDS strategic plan 2020-2023  National health strategic plan 2020 – 2030  National malaria strategic plan 2016 – 2020  National laboratory strategic plan 2021 – 2025  National human resources for health 2021  National TB strategic plan 2024 – 2028  National strategic plan for universal health coverage 2020 - 2030  National strategic plan for the development of health laboratory services 2021 – 2025  National malaria strategic plan 2020 – 2023  National strategic plan for the control of TB 2024 - 2028  National strategic plan for the control of non-communicable diseases 2016  National strategic plan a multisectoral vision for family planning 2021 - 2025  National integrated strategic plan for reproductive maternal, newborn, child, adolescent health and nutrition health 2019 - 2022  National Covid19 outbreak preparedness and response plan 2020  National strategic plan for the development of health laboratory services 2021 - 2025 |
| Eswatini | National HIV/AIDS national strategic plan 2018-2023  National laboratory strategic plan 2021-2023  National health sector strategic plan 2025 - 2028  National multisectoral HIV/AIDS strategic framework 2018 - 2023  National strategic plan for the prevention and control of non-communicable diseases 2021 – 2023  National strategic plan for tuberculosis 2024 - 2028 |
| Ethiopia | National strategic plan for TB and leprosy control 2014 – 2020  National non-communicable strategic plan 2018 - 2025  Strategic plan for health laboratory system in Ethiopia 2023 - 2027  National strategic plan for HIV/AIDS 2021 – 2025  National Essential Diagnostics List 2024  Covid19 multi sectoral preparedness and response plan 2020  National reproductive health strategic plan 2021 – 2025  National strategy for newborn and child survival in Ethiopia 2016 - 2020  National malaria elimination strategic plan 2021 – 2025  National equity health strategic plan 2021 – 2025  National one health strategic plan 2018 – 2022  Health sector transformation plan II 2021 – 2025  National health sector strategic plan for early childhood development 2021 - 2025 |
| Gabon | National malaria strategic plan 2018-2021  National health strategic plan 2024 - 2028  National TB strategic plan 2024 – 2028  National laboratory policy 2023 – 2033  National laboratory strategic plan 2023 - 2027 |
| Kenya | National health policy 2014-2030 (*draft*)  National laboratory services strategic plan 2023/24-2027/28 (draft)  National laboratory strategic plan 2016-2020  National AIDS strategic plan framework II 2021 - 2025  Non communicable disease national strategic plan 2021-2025  National malaria strategic plan 2019-2023  National TB strategic plan 2019-2023  National point of care testing implementation roadmap 2019  Integrated diagnostic network optimization 2023  National Essential diagnostics list 2023  Kenya Essential Medical Commodities List (KEMCL)  Reproductive, maternal, newborn child and adolescent investment framework 2016  Newborn and child health strategic plan 2021 – 2025  Covid19 policy response 2020  State department for public and professional standards, strategic plan 2023 – 2027  National community health strategy 2020 - 2025 |
| Malawi | TB and leprosy national strategic plan 2021-2025  National HIV/AIDS strategic plan 2020 – 2025  National HIV/AIDS policy 2022 - 2027  National health policy 2017  National laboratory services policy 2023 – 2030  National laboratory services operational plan 2023 - 2030  National health community strategy 2017 – 2022  National community health framework 2023 - 2030  National laboratory strategic plan (2017-2022)  National essential diagnostics list 2023  National action plan for the prevention and management of NCD 2017 - 2022  National malaria strategic plan 2023- 20230  National Covid19 preparedness and response strategy and plan 2021  National POC testing implementation guidelines 2012  National HIV POCT implementation framework 2017  National sexual and reproductive health and rights strategy 2021 - 2025 |
| Mozambique | National health sector strategic plan 2020 - 2024  National strategy for clinical laboratories 2020-2024  Organization and operation standards for clinical and public health laboratories 2025  National HIV/AIDS strategic plan 2021-2025  National malaria strategic plan 2017 - 2022  National non-communicable disease strategic plan 2020 - 2029  National strategic plan to end TB 2023 – 2030  National Plan for the Triple Elimination of Mother-to-Child Transmission of HIV, Syphilis and Hepatitis B 2020-2024  National Essential Diagnostics List (*draft*)  Covid19 preparedness response plan 2021 |
| Nigeria | National guideline for the integration of medical laboratory services and systems 2024  National medical laboratory strategic plan 2023-2027  National medical laboratory policy 2023  National essential diagnostic list  National multi-sectoral action plan for the prevention and control of NCD (2019 – 2025)  Second National health strategic plan 2018 – 2022  National HIV/AIDS strategic plan 2023 – 2027  National HIV/AIDS strategic framework 2021 - 2025  National malaria strategic plan 2021 – 2025  National strategic plan for TB control 2021 – 2026  National TB laboratory operational plan 2024 – 2026  National task-shifting and task sharing policy for essential health care services 2014  National guideline for the integration of medical laboratory services and systems 2024  National reproductive, maternal, newborn, child, adolescent, elderly health plus nutrition quality of care monitoring, evaluation, accountability and learning (MEAL) plan 2022 - 2027 |
| Siera-Leone | National laboratory strategic plan 2022 - 2026  National strategic plan for HIV/AIDS 2020  National TB strategic plan 2016 – 2020  National health sector strategic plan 2021 – 2025  National reproductive, maternal, newborn, child and adolescent health strategic plan 2017 – 2021  Consolidated guidelines on the HIV prevention, diagnosis, treatment and care 2023  National strategic plan for non-communicable diseases 2020 – 2024  Covid19 emergency preparedness and response plan 2020  National community health worker strategic policy 2021  National malaria control strategic plan 2016 - 2020 |
| South Africa | National health strategic plan 2021 – 2025  National malaria strategic plan 2019 – 2023  National non-communicable disease strategic plan 2022 – 2027  National health laboratory strategic plan 2020 – 2025  National strategic plan for HIV, TB and STIs 2023 – 2028  National TB strategic plan 2023 - 2028  National human resources for health strategy 2020 – 2030  Implementation of point-of-care testing policy (*draft 2020*)  Point of care testing implementation plan (*draft 2025*)  Covid19 preparedness and response plan 2020  National maternal, newborn, child and women’s health and nutrition strategic plan 2016 |
| South Sudan | National health policy 2016-2026  National health sector strategic plan 2023-2027  National laboratory policy 2019  National laboratory strategic plan 2024-2028  National POC testing multi-disease diagnostic implementation framework 2021  National certification framework for rapid HIV testing 2021  National malaria strategic plan 2021 – 2025  National essential diagnostic list 2024  National TB strategic plan 2020 – 2024  National STI strategic plan 2018-2022  Every newborn action plan 2018 - 2022  National non communicable disease strategic plan 2010 - 2015  National certification framework for HRT 2021  Covid19 preparedness and response operation 2021  Maternal, newborn child and adolescent health and nutrition 2020 -2024 |
| Tanzania | National health sector strategic plan 2021-2026  National essential diagnostics list (draft) 2023  National HIV/AIDS strategic plan 2017-2022  National HIV/AIDS strategic plan 2024-2027  National laboratory strategic plan 2016-2021  National malaria strategic plan 2021-2025  Non communicable diseases national strategic plan 2016-2020  Non communicable diseases national strategic plan 2021-2026  TB and leprosy national strategic plan 2015-2020  National TB and leprosy national strategic plan 2020 - 2025  National framework for POC testing certification 2017  East African community Covid19 response plan 2020  National strategic plan to improve reproductive, maternal, newborn, child and adolescent health in Tanzania 2016 – 2020 |
| Uganda | Ministry of health strategic plan 2021 - 2025  National laboratory strategic plan 2021-2025  National health laboratory services policy 2017  National strategic plan for TB and leprosy 2020-2025  National malaria reduction strategy 2014-2020  National HIV/AIDS strategic plan 2021 - 2025  National multisectoral strategic plan for the prevention and control of NCD 2018 – 2023  National Covid19 preparedness and response plan 2020 – 2021  National reproductive maternal, newborn, child adolescent and healthy aging 2023 – 2027 (sharpened plan and investment case II)  National community health strategy 2022 |
| Zambia | National health strategic plan 2022-2026  National biomedical laboratory strategic plan 2023-2027  National biomedical laboratory strategic plan 2018-2022  Malaria operational plan 2022-2024  National HIV/AIDS strategic framework 2017-2021  National HIV/AIDS strategic framework 2023 - 2027  National non-communicable disease strategic plan 2013 – 2016  National HIV POC certification framework 2019  National community health strategy 2022 – 2026  National biomedical laboratory policy 2023 |
| Zimbabwe | National health strategy 2021-2025  National health laboratory strategic plan 2022-2026  Medical laboratory and POC testing national certification framework 2024  National HIV and STI strategy 2021-2025  National HIV/AIDS strategic plan 2021-2025  Operational service and delivery manual for prevention, care and treatment of HIV 2022  POC testing guideline 2016-2020  National strategic plan for TB 2017 – 2020  Covid19 preparedness and response plan 2020 |
